# Epstein–Barr Virus Infection and Gastric Cancer: A Systematic Review

**DOI:** 10.1097/MD.0000000000000792

**Published:** 2015-05-22

**Authors:** Xin-Zu Chen, Hongda Chen, Felipe A. Castro, Jian-Kun Hu, Hermann Brenner

**Affiliations:** From the Division of Clinical Epidemiology and Aging Research, German Cancer Research Center (DKFZ), Heidelberg, Germany (X-ZC, HC, FAC, HB); Department of Gastrointestinal Surgery (X-ZC, J-KH); Laboratory of Gastric Cancer, State Key Laboratory of Biotherapy, West China Hospital, Sichuan University, Chengdu, China (X-ZC, J-KH); and German Cancer Consortium (DKTK), Heidelberg, Germany (HB).

## Abstract

Epstein–Barr virus (EBV) infection is found in a subset of gastric cancers. Previous reviews have exclusively focused on EBV-encoded small RNA (EBER) positivity in gastric cancer tissues, but a comprehensive evaluation of other type of studies is lacking.

We searched the PubMed database up to September, 2014, and performed a systematic review.

We considered studies comparing EBV nucleic acids positivity in gastric cancer tissue with positivity in either adjacent non-tumor tissue of cancer patients or non-tumor mucosa from healthy individuals, patients with benign gastric diseases, or deceased individuals. We also considered studies comparing EBV antibodies in serum from cancer patients and healthy controls.

Selection of potentially eligible studies and data extraction were performed by 2 independent reviewers. Due to the heterogeneity of studies, we did not perform formal meta-analysis.

Forty-seven studies (8069 cases and 1840 controls) were identified. EBER positivity determined by in situ hybridization (ISH) was significantly higher in cancer tissues (range 5.0%–17.9%) than in adjacent mucosa from the same patients or biopsies from all control groups (almost 0%). High EBV nuclear antigen-1 (EBNA-1) positivity by PCR was found in gastric cancer tissues, but most were not validated by ISH or adjusted for inflammatory severity and lymphocyte infiltration. Only 4 studies tested for EBV antibodies, with large variation in the seropositivities of different antibodies in both cases and controls, and did not find an association between EBV seropositivity and gastric cancer.

In summary, tissue-based ISH methods strongly suggest an association between EBV infection and gastric cancer, but PCR method alone is invalid to confirm such association. Very limited evidence from serological studies and the lack of novel antibodies warrant further investigations to identify potential risk factors of EBV for gastric cancer.

## Abstract 2

Supplemental Digital Content is available in the text

## INTRODUCTION

Gastric cancer is the third most common cause of cancer death worldwide, with >700,000 deaths estimated to have occurred in 2012.^1^ Gastric carcinogenesis is thought to be associated with multiple environmental and genetic factors. Among environmental factors, infection with the bacterium *Helicobacter pylori* is an established main risk factor.^2,3^ However, increasing evidence suggests that a subset of gastric cancers is associated to Epstein–Barr virus (EBV) infection.^4–6^ Recent cancer genome atlas research has provided a molecular classification defining EBV-positive gastric cancer as a specific subtype.^7^

EBV can be found in the malignant epithelial cells of gastric adenocarcinomas.^8–10^ Positivities and characteristics of the EBV-positive cancers have been summarized previously (supplementary Table 1, http://links.lww.com/MD/A257).^11–16^ However, the positivity of EBV infection in normal gastric mucosa, and other gastric diseases, such as dyspepsia, gastritis and peptic ulcer, is largely unexplored.^17^ A recent study found all normal gastric mucosa samples from healthy individuals EBV RNA-negative, whereas positivity was 46% in tissues with gastritis, with frequent infiltration of EBV infection.^17^ These patterns suggest that EBV infection might be associated with induction of persistent gastric mucosa inflammation and subsequent carcinogenesis.

In this systematic review, we aim to provide a comprehensive overview on published epidemiological studies based on in situ hybridization (ISH), polymerase chain reaction (PCR) or serology, comparing EBV nucleic acids positivity in gastric cancer tissues and in adjacent non-tumor tissues; EBV nucleic acids positivity in gastric cancer tissues and in non-tumor mucosa from healthy individuals, patients with benign gastric diseases, or deceased individuals; and EBV seropositivity among gastric cancer patients and healthy controls.

## METHODS

### Search Strategy

The PubMed database was searched up to September 14, 2014, using the following search algorithm (“stomach neoplasms” [MeSH Terms] OR (“stomach” [All Fields] AND “neoplasms” [All Fields]) OR “stomach neoplasms” [All Fields] OR (“gastric” [All Fields] AND “cancer” [All Fields]) OR “gastric cancer” [All Fields]) AND (EBV [All Fields] OR (“EB” [All Fields] AND “virus” [All Fields]) OR “EB virus” [All Fields] OR “herpes virus 4, human” [MeSH Terms] OR “human herpes virus 4” [All Fields] OR (“epstein” [All Fields] AND “bar” [All Fields] AND “virus” [All Fields]) OR “epstein bar virus” [All Fields])) NOT (“animal” [Filter]). The search was limited to studies in humans.

### Studies Included

Our review focused on studies including patients with histologically proven primary gastric adenocarcinoma. Studies addressing gastric lymphoma, gastric lymphoepithelioma-like cancer, gastrointestinal stromal tumor, remnant stomach cancer, or cardia squamous cell carcinoma were excluded due to potential differences in carcinogenesis. There was no limitation on cancer stage and treatment strategy.

Studies were included if they also reported on EBV positivity in adjacent tumor tissue and /or non-gastric cancer controls. Controls included patients from outpatient or inpatient settings including patients who died from nonmalignant diseases, or subjects from the general population. Non-malignant diseases included non-ulcer diseases (NUDs) concerning intestinal metaplasia, dysplasia, atrophic gastritis, adenoma, and polyp etc, as well as peptic ulcer diseases (PUDs).

### EBV Status

We included studies that evaluated the presence of EBV in tissues (endoscopic biopsy tissues, resected cancer tissues, or postmortem gastric mucosa) and in serum samples (peripheral blood samples). Laboratory methods for EBV were ISH or PCR for resected tissue, biopsy, or blood; and enzyme-linked immunosorbent assay (ELISA) or immunofluorescence assay (IFA) for serum samples.

Target markers for EBV included: EBV-encoded small RNA (EBER)-1 or -2 for ISH; Epstein–Barr nuclear antigen (EBNA)-1, EBV Bam M fragment (Bam-M), EBV Bam HI W fragment (BamHI-W) for PCR; EBNA, EBV viral capsid antigen (VCA), EBV diffuse early antigen (EA-D), and EBV restricted early antigen (EA-R) for serology.

### Selection of Publications and Data Extraction

Potentially eligible studies were selected by 2 independent reviewers (X-ZC and HC). The primary selection was performed by browsing the titles and abstracts. Potentially eligible studies underwent full text review. References of identified studies were additionally screened for potentially missed studies. Potential discrepancies in study selection were resolved by further review and discussion with Castro F.A.

The data extraction was likewise carried out independently by 2 reviewers (X-ZC and HC). Extracted items included general study characteristics (year, country, study design), characteristics of the study populations (size, sex, age, disease-related factors), and types of measurements (specimen types, analytic procedures). Number of cases and controls were extracted from all publications or in few cases calculated from the reported percentage of cases. Potential discrepancies in extracted items if any were resolved by further review and discussion by Castro F.A.

### Statistical Analysis

Study-specific odds ratios (ORs) and their 95% confidence intervals (CIs) were calculated where applicable by MedCalc software version 12.7.4 (http://www.medcalc.org/calc/odds_ratio.php). Due to the heterogeneity of studies, we did not perform formal meta-analysis.

### Ethical Review and Reporting

This systematic review worked with the literature and did not directly involve human beings or animals, and therefore was not submitted for any ethical approval. This study is reported according to the Preferred Reporting Items for Systematic Reviews and Meta-Analyses (PRISMA) statement.^18^

## RESULTS

### Literature Search

**FIGURE 1:**
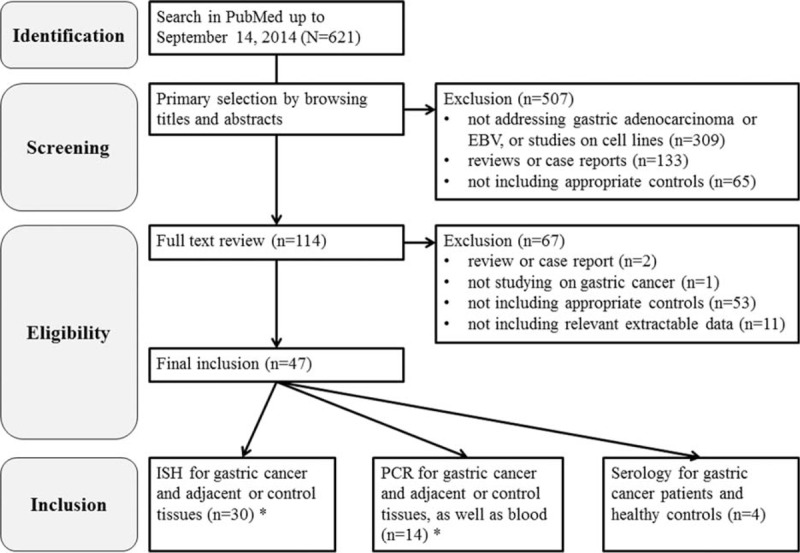
Flow chart of literature selection. (^∗^The 2 tests contain an overlapping study).

The flow chart of the literature search is shown in Figure 1. Three population-based and 44 hospital-based case–control or cross-sectional studies were eligible for inclusion in the systematic review.^5,19–64^ Among selected studies, 34 studies compared gastric cancer and any kind of non-cancer tissue by ISH method; 13 studies compared tissue of cancer patients and any non-cancer control by PCR method, as well as blood samples were tested in 1 study; and 4 studies compared serum samples from cancer and healthy controls by serological measurements. In total, 9909 individuals (8069 cases and 1840 controls) were included in present systematic review. Detailed information on the selected studies is shown in supplementary Tables 2 to 4, http://links.lww.com/MD/A257.

### Detection of EBV Infection by ISH

Thirty-four studies compared gastric cancer tissue to any kind of control tissues by ISH approach to testing EBER-1 or -2 (Table 1).^5,20,22–27,29,31–34,37–41,43–47,49–53,57,58^ The positivity of EBV RNA in cancer cells ranged from 5.0% to 17.9% by ISH. In contrast, in most studies, all of adjacent non-cancer tissues were consistently negative for EBV RNA in epithelial cells, or had positivity close to zero, with the exception of 2 studies of Fukayama et al^23^ and Shousha et al,^26^ which reported high positivity in adjacent non-cancer tissue (35.3% and 58.3%, respectively). Likewise, none of the samples from the gastric ulcer or normal gastric mucosa of deceased patients was EBER-positive.

**TABLE 1:**
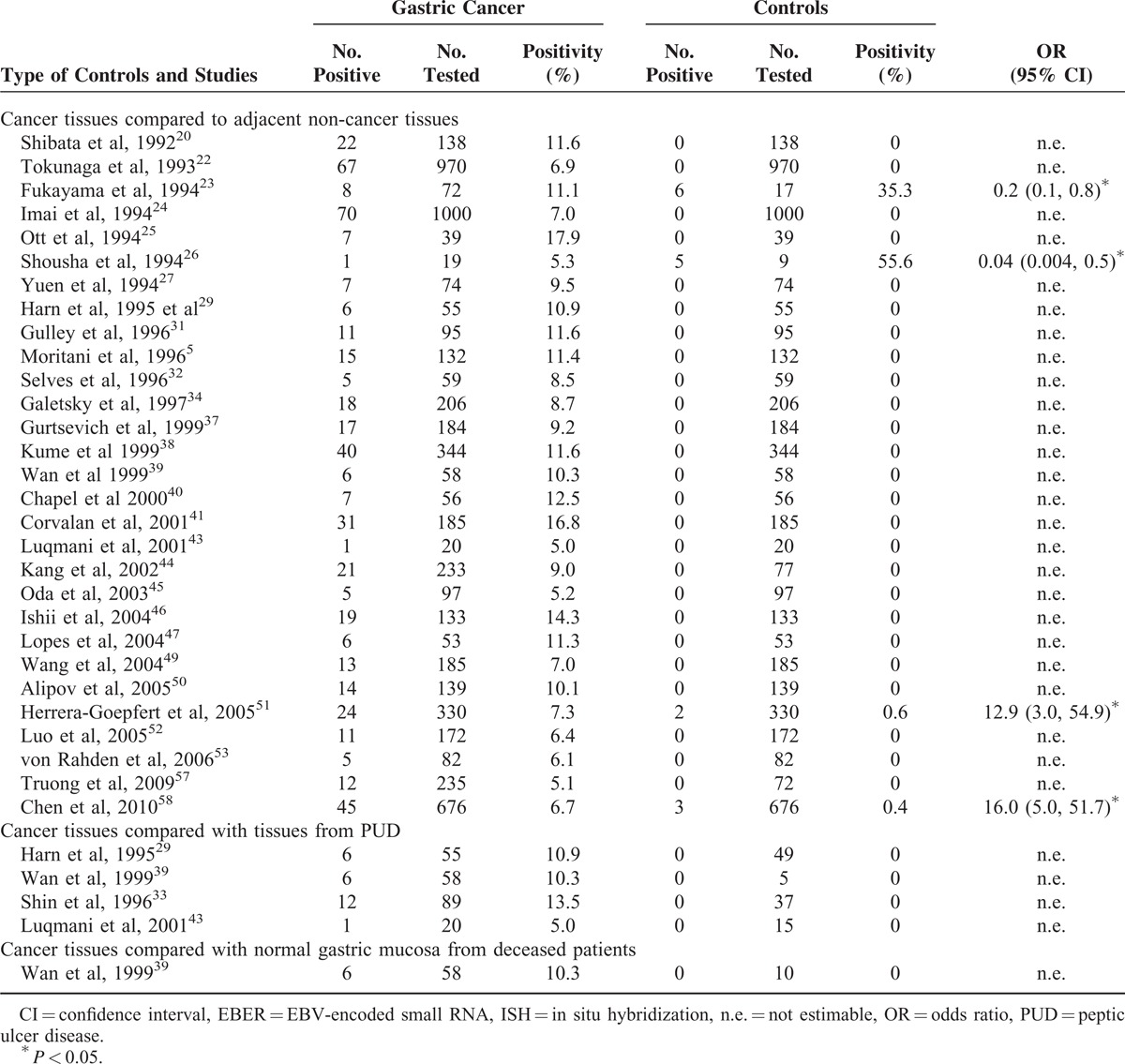
Comparison of EBV Positivity Between Gastric Tumor Tissue and Any Controls By ISH for EBER

### Detection of EBV Infection by PCR

A total of 13 studies (Table 2)^21,28,35,36,42,45,48,55,59,60–64,^ compared the EBV nucleic acids (EBNA-1, -2, Bam-M, and BamHI-W) between gastric cancer tissues and any non-cancer tissues, as well as one study that compared EBV BamHI-W in blood between gastric cancer patients and healthy controls. The positivities of EBNA-1 and BamHI-W fragments in tissue samples from cancer patients was usually significantly higher than those in biopsies from any kind of control groups, with the exception of the study of de Aquino et al^60^ when compared with adjacent non-cancer tissues. Additionally, in the study of Yuan et al,^63^ all gastric cancer tissues, adjacent non-cancer tissues, and biopsies from patients with NUD were negative. However, positivities of EBV nucleic acids tested by PCR methods varied substantially in both cases and controls. Extremely high positivities of ≥80% were found in 3 studies from India, which tested EBNA-1 in gastric cancer tissues.^55,59,61^ Additionally, 2 studies of de Aquino et al^60^ and Durmaz et al^35^ testing Bam-M and EBNA-2, respectively, found the positivities were 50% to 60% in gastric cancer tissues. The only study that tested EBV BamHI-W in blood found the positivities were 35.5% and 3.6% among gastric cancer patients and healthy controls, respectively (OR = 14.8, 95% CI 5.7–38.2).^42^

**TABLE 2:**
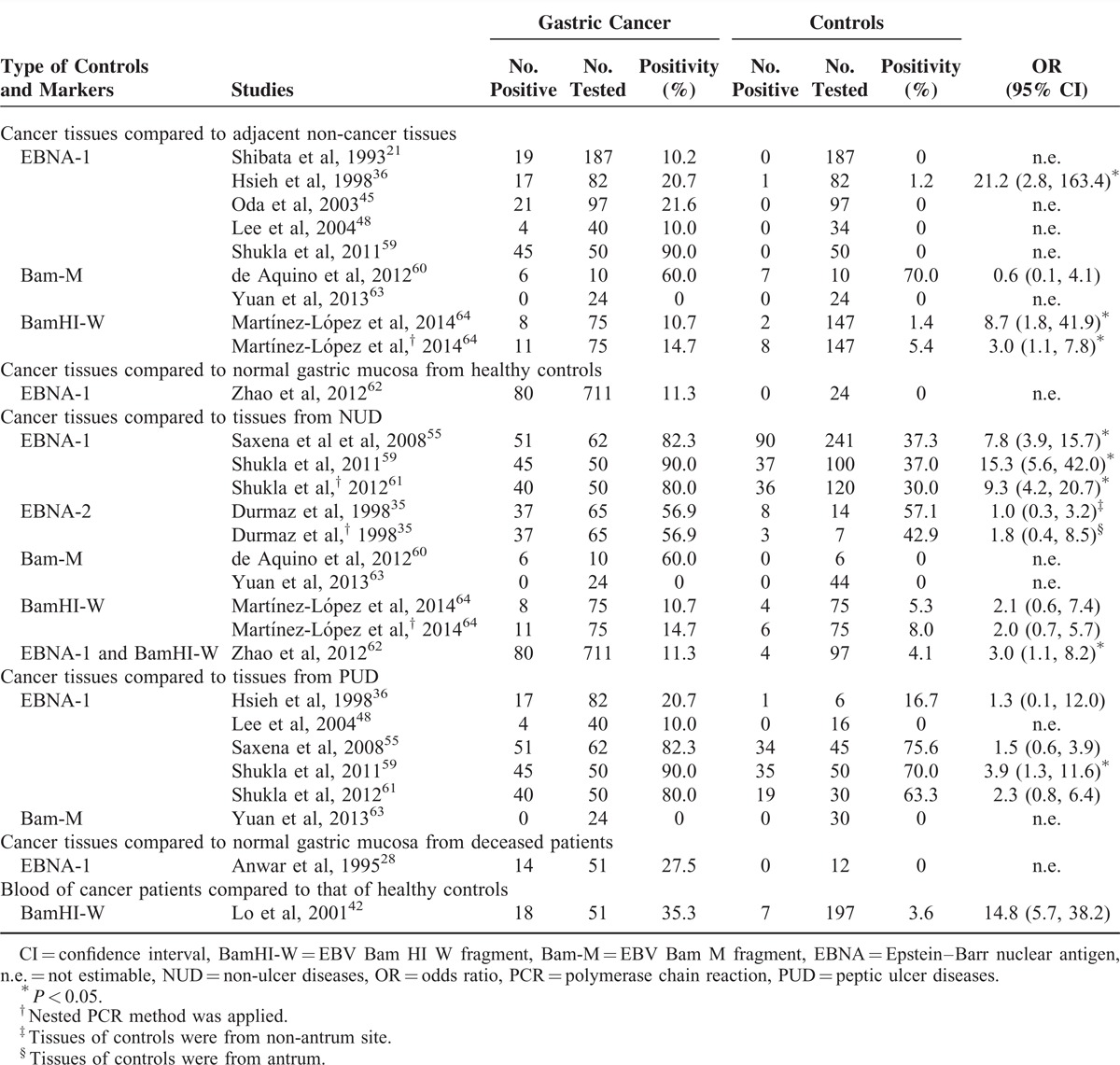
Comparisons of EBV Positivity in Tumor Tissue of Gastric Cancer Patients and Any Controls By PCR Method

### Serology of EBV Infection for Gastric Cancer

Only 4 studies compared results of EBV serology between gastric cancer patients and healthy individuals (Table 3).^19,30,54,56,^ These studies used ELISA or IFA to test for antibodies against one or several EBV antigens, including EBNA, VCA, or EA (EA-D or EA-R). The distribution of EBV seropositivity in gastric cancer patients and healthy controls varied across most studies and antibodies. EBNA IgG and VCA IgG had higher seropositivities among both cancer patients and healthy controls than other antibodies. Only EBNA IgG was slightly less frequent in cancer patients than in controls in 2 studies, although differences were not statistically significant. VCA IgG appeared consistently more frequent in cancer patients than in controls, but only 1 Japanese study from 1991 had reported a significant difference in the EBV seropositivity between cases and controls, using IFA to detect VCA IgG at the cutoff >1:640 (OR = 22.2, 95% CI 7.8–63.1).^19^ Additionally, inconsistent results and absence of differences between cases and control were reported for VCA IgA, EA IgG, EA-D IgG, and EA-R IgG.

**TABLE 3:**
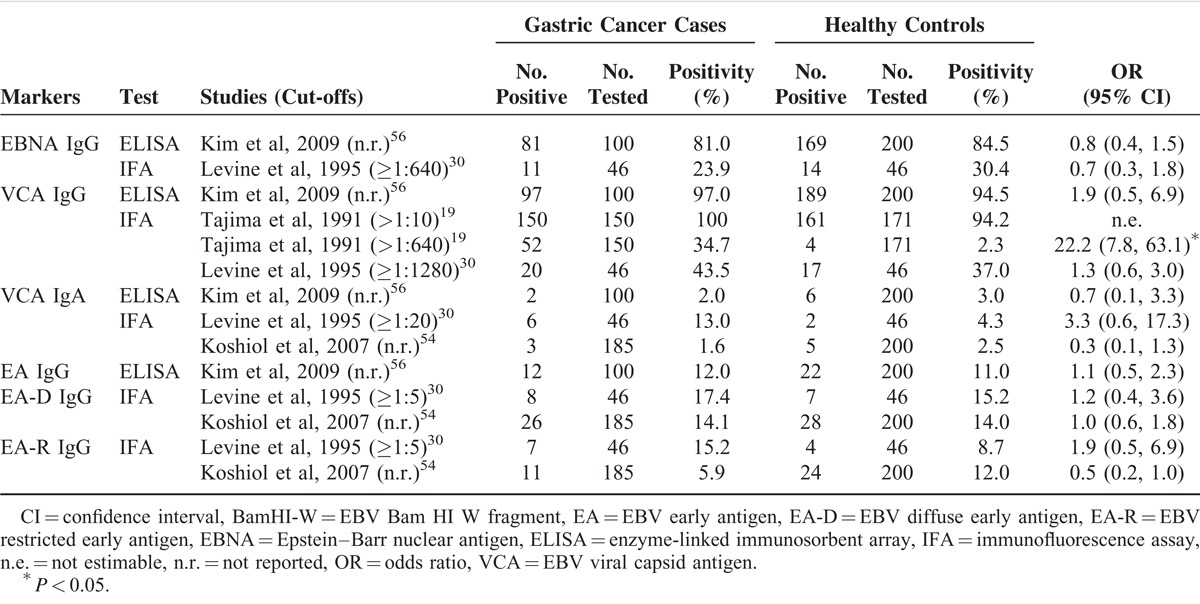
Comparisons of EBV Seropositivity Between Gastric Cancer Patients and Healthy Controls

## DISCUSSION

To our knowledge, this is the first comprehensive systematic review of epidemiological studies on the association between EBV infection and gastric cancer. EBER positivity by ISH ranged from 5.0% to 17.9% in gastric cancer tissues, but was rare in both adjacent non-cancer tissues and gastric biopsies of healthy controls or patients with benign gastric diseases (almost 0%). Additionally, we found positivities of EBNA-1 and BamHI-W by PCR to be consistently higher in tissues or blood from gastric cancer patients than in any non-cancer sample, and positivity tended to be associated with the local inflammatory severity. Studies evaluating the seropositivity of EBV antibodies were scarce and the evidence for each of the tested antigens was inconsistent across studies and not significantly different between gastric cancer patients and healthy controls.

Previous reviews and meta-analyses have been exclusively focused on the positivity and characteristics of EBV-positive gastric cancers by ISH only. Summarized results from 6 systematic reviews, meta-analyses, or pooled analyses are shown in supplementary Table 1, http://links.lww.com/MD/A257. Our findings agree with previous meta-analyses that reported an overall EBV RNA positivity from 6.9% to 8.8%.^11–16^ Previous meta-analyses also showed that EBV-positive gastric cancers are more common among males, younger patients and those localized mainly at the cardia and body of the stomach, as well as those with postgastrectomy remnant stomach.

Detection of EBV RNA in gastric cancer tissue by itself does not provide sufficient evidence to establish a causal role of EBV in gastric carcinogenesis. An additional evidence for such a role would be differences in EBV RNA prevalence between cancer and non-cancer tissue by ISH method, evaluated in this review. Despite the heterogeneity in study designs and results, an important observation seems to support the association of EBV with gastric carcinogenesis: evidence obtained from studies using gold standard tissue methods, such as ISH, demonstrated that most of the adjacent non-cancer tissues and biopsy samples from healthy individuals or patients with benign gastric diseases were EBER-negative. The consistently negative existence in epithelial cells of such internal controls (adjacent mucosa) and external controls (mucosa from healthy person or patient with benign disease) can inversely evidence that EBV infection is a risk factor for gastric cancer. However, nasopharyngeal carcinoma, another epithelial tumor caused by EBV, has been shown as monoclonal proliferation of a single EBV-infected progenitor epithelial cell.^65^ Viral monoclonality in EBV-positive gastric cancer samples is arguably the strong evidence of a causal relationship between EBV infection and gastric cancer development.^24,25^ Additionally, for EBV-positive gastric cancer, several associated genetic alterations can be displayed through genome atlas research, including recurrent PIK3CA mutations, extreme DNA hypermethylation, and amplification of JAK2, CD274, and PDCD1LG2.^7^ They might be critical understandings of molecular mechanism of EBV-associated gastric carcinogenesis.

In contrast, PCR methods are more sensitive but less specific than gold standard ISH method. However, based on PCR tests, an additional observation was a suggestion of a gradient in the EBV infection among the control groups and gastric cancer patients. Positivities of EBNA-1 increased from 0% in a healthy control group, 4.1% to 37.3% in patients with non-ulcer gastric diseases, to 16.7% to 75.6% in patients with peptic ulcer diseases. This observation is mainly based on 3 studies, in which EBNA-1 positivity was extremely high in cancer samples (80%–90%). Thus cross-contamination picked up by PCR methods cannot be ruled out. Another more important explanation of the gradient trend among non-cancer tissues and the difference between gastric cancer and noncancer tissues needs to be underlined. PCR method is invalid to distinguish cancer cells with lymphocytes infiltrating in cancer stromal, and therefore it is not possible to know from where the EBV nucleic acids are amplified. Vast majority of people are EBV carriers (around 90%), and lymphocytes are possibly infected with EBV and contain EBV nucleic acids.^66^ With progression of local inflammation, the amount of lymphocytes infiltrating inside or around solid tumor can be increased, whereas obvious lymphocyte infiltration is frequently presented in cancer tissues.^67,68^ Besides, another argument is that EBER-positive lymphocytes can be labeled inside or around gastric cancer tissues by ISH method.^21,22^ The increased and high positivity of EBNA-1 by PCR might be a reflection of inflammatory severity and amount of infiltrating lymphocytes, instead of the difference in amount of cancer and epithelial cells infected with EBV. However, this hypothesis is not enough convincing and a confirmative conclusion is unable to be suggested based on above evidence. First, study involving health controls comprised from only one study.^62^ NUD such as intestinal metaplasia and dysplasiais is not always related to inflammation even compared with healthy controls. Furthermore, PUD patients usually have high *Helicobacter pylori* infection rate, whereas the local inflammation is therefore mainly due to the co-infection of *Helicobacter pylori* instead of EBV. Besides, 1 study still showed 0% of EBNA-1 positivity among PUD patients.^48^ Therefore, if PCR method is used, it should be interpreted with caution and better to be further validated by using ISH method. Furthermore, it is necessary that PCR results should be also adjusted by lymphocyte infiltration.

Serological markers for EBV have been suggested to be useful to evaluate cumulative lifetime exposure and reactivation of the viral infection. EBNA IgG and VCA IgG can retain at high level in the life time after acute stage of EBV infection. In nasopharyngeal carcinomas, EBV-specific IgA serum antibodies, specially, EA and VCA IgA, were suggested to be able to identify individuals at early stage of the disease and also potential predictors of disease prognosis.^69,70^ We identified only 4 studies comparing EBV seropositivity between gastric cancer patients and healthy controls, all of which were conducted among all Eastern Asians or Eastern Asian descendants. With the exception of a study of Tajima et al in 1991, all studies used a matched design, included prospective samples collected several years prior to cancer diagnosis, and showed no significant difference in the EBV antibody levels between cases and controls. In contrast, the Japanese study found that VCA IgG antibodies titers were significantly higher among gastric cancer patients (34.7% in cases and 2.3% in controls); however, serum samples were collected after diagnosis of gastric cancer, and no matching method was mentioned.^19^ Shinkura et al^71^ compared seropositivity of EBV-specific antibodies among EBV-positive and -negative gastric cancers and healthy controls. It was found that VCA IgA and EA IgG had higher seropositivity among EBV-positive gastric cancers than those among EBV-negative gastric cancers. The seropositivity of EA-IgG was higher among EBV-negative gastric cancers than that among healthy controls. Additionally, Shatter et al found significantly higher geometric mean antibody titers for both VCA and EBNA among subjects with dysplasia compared with those with gastritis or intestinal metaplasia, and therefore suggested a possible role for EBV reactivation at an early phase of gastric carcinogenesis.^72^

Seroepidemiological data on gastric cancer are still very limited and it is not clear whether similar patterns of antibodies against EBV as those observed for nasopharyngeal cancer might also apply to gastric cancer. An important difference between gastric cancer and nasopharyngeal cancer is that merely a minority of gastric cancer cases are associated with EBV infection.^73^ Moreover, seropositivity of EBV antibodies reflects the life-time infection of EBV acquired from childhood, but it may not distinguish the EBV-associated gastric cancer patients with healthy population because of the high prevalence of EBV antibodies among population. As a result, interpretation of EBV seropositivity remains a challenge. In this case, comparison on the titers of EBV antibodies between gastric cancer patients and healthy controls might additionally inform to judge the association of EBV infection with gastric cancer risk. Furthermore, novel antibodies against EBV-specific antigens are also expected to assess the association between EBV seropositivity and gastric cancer risk.

Nevertheless, aforementioned 3 techniques have different defects in identifying a high-risk population for EBV-associated gastric cancer. ISH test is a reliable measurement, but requires invasive and complex techniques. Additionally, EBERs are always negative in non-cancer gastric mucosa from both biopsies of cancer-free individuals and adjacent normal stomach of gastric cancer. Therefore, these 2 reasons make ISH invalid to screen a high-risk subpopulation for EBV-associated gastric cancer. PCR based on tissues is also an invasive test, but a likely dose–response correlation between EBNA-1 positivity and inflammatory severity, which is possibly confounded by *Helicobacter pylori*–associated local inflammation and lymphocyte infiltration at mucosa. Further understanding on the interaction between EBV and *Helicobacter pylori* infection is required. PCR for BamHI-W fragment based on blood was suggested to be a risk factor in only 1 study. Sample size was also very small in the single study reporting on a major difference in BamHI-W positivity determined in blood between cases and controls. This interesting result reported in 2001 seems not to have been replicated since then. More studies need to be repeated to confirm the association before employing BamHI-W in screening EBV-associated gastric cancer. A classical epidemiological study on identifying a high-risk population is commonly based on serology of specific antibodies. However, a critical limitation of serological studies is that EBV infections are widespread, >90% of the adult population have had some exposure to the infection at some time of their life and carry the corresponding antibody signatures. For example, Kim et al^56^ found the seropositivities of VCA IgG and EBNA IgG were as high as 94.5% and 84.5%, respectively, in the healthy controls. In such a situation of very high population prevalence, it may not be possible to find relevant difference in seropositivity between cases and controls. In particular, a more interesting or relevant question about EBV serology in this context might be whether virulence markers of the virus or susceptibility markers of the host can be identified that would allow identification of risk group for developing gastric cancer. Although specific viral antigens were addressed in some of the studies, sample sizes were mostly very small, which makes it difficult to draw firm conclusions.

Several other limitations of our review deserve careful discussion. We were unable to provide summary estimates on the association of EBV infection and gastric cancer because existing studies differed greatly in their study population, laboratory methods, and control selection. Likewise, many studies did not report adequate information on cancer site and other morphological features. A major obstacle in the evaluation of a possible etiological role of EBV in gastric cancer is the lack of prospective studies that hinders ruling out reverse causality. Serological markers may provide an opportunity to evaluate previous exposure, but published evidence is still very sparse. Currently, there is no an ideally epidemiological approach to further evaluate the suggested causal relationship or association of EBV infection and gastric cancer. The discrepancy between epidemiological analysis and molecular biological or virological observation needs to be dissolved with novel epidemiological analysis based on reliable molecular analysis.

In conclusion, evidence based on ISH method strongly suggests an association between EBV infection and gastric cancer risk, but PCR method alone is invalid to confirm such association. Very limited evidence from serological studies and the lack of novel antibodies warrant further investigations to identify potential risk factors of EBV for gastric cancer.

## References

[1] Ferlay J Soerjomataram I Ervik M Dikshit R Eser S Mathers C Rebelo M Parkin DM Forman D Bray F GLOBOCAN 2012 v1.0, Cancer Incidence and Mortality Worldwide: IARC CancerBase No. 11 [Internet]. Lyon, France: International Agency for Research on Cancer 2013: Available from: http://globocan.iarc.fr/Pages/fact_sheets_population.aspx.

[2] Wroblewski LE Peek RM Jr Helicobacter pylori in gastric carcinogenesis: mechanisms. *Gastroenterol Clin North Am* 2013; 42:285–298. 23639641 10.1016/j.gtc.2013.01.006 PMC3648881

[3] Gao L Michel A Weck MN Helicobacter pylori infection and gastric cancer risk: evaluation of 15 H. pylori proteins determined by novel multiplex serology. *Cancer Res* 2009; 69:6164–6170. 19602590 10.1158/0008-5472.CAN-09-0596

[4] Burke AP Yen TS Shekitka KM Lymphoepithelial carcinoma of the stomach with Epstein-Barr virus demonstrated by polymerase chain reaction. *Mod Pathol* 1990; 3:377–380. 2163534

[5] Moritani S Kushima R Sugihara H Phenotypic characteristics of Epstein-Barr-virus-associated gastric carcinomas. *J Cancer Res Clin Oncol* 1996; 122:750–756. 8954173 10.1007/BF01209123 PMC12200040

[6] Akiba S Koriyama C Herrera-Goepfert R Epstein-Barr virus associated gastric carcinoma: epidemiological and clinicopathological features. *Cancer Sci* 2008; 99:195–201. 18271915 10.1111/j.1349-7006.2007.00674.x PMC11158035

[7] Cancer Genome Atlas Research Network. Comprehensive molecular characterization of gastric adenocarcinoma. *Nature* 2014; 513:202–209. 25079317 10.1038/nature13480 PMC4170219

[8] Ohfuji S Osaki M Tsujitani S Low frequency of apoptosis in Epstein-Barr virus-associated gastric carcinoma with lymphoid stroma. *Int J Cancer* 1996; 68:710–715. 8980171 10.1002/1097-0215(19961211)68:6<710::aid-ijc2910680602>3.0.co;2-6

[9] Vasef MA Weiss LM Chen YY Gastric lymphoepithelioma-like carcinoma and jejunal B-cell MALT lymphoma with large cell transformation. Demonstration of EBV with identical LMP gene deletions in the carcinoma and large cell lymphoma. *Am J Clin Pathol* 1996; 105:560–566. 8623763 10.1093/ajcp/105.5.560

[10] Harn HJ Ho LI Chung WH Epstein-Barr virus-associated typical gastric carcinoma detected by in situ hybridization and polymerase chain reaction. *J Clin Gastroenterol* 1995; 20:253–254. 7797839 10.1097/00004836-199504000-00020

[11] Sousa H Pinto-Correia AL Medeiros R Epstein-Barr virus is associated with gastric carcinoma: the question is what is the significance? *World J Gastroenterol* 2008; 14:4347–4351. 18666324 10.3748/wjg.14.4347 PMC2731187

[12] Lee JH Kim SH Han SH Clinicopathological and molecular characteristics of Epstein-Barr virus-associated gastric carcinoma: a meta-analysis. *J Gastroenterol Hepatol* 2009; 24:354–365. 19335785 10.1111/j.1440-1746.2009.05775.x

[13] Murphy G Pfeiffer R Camargo MC Meta-analysis shows that prevalence of Epstein-Barr virus-positive gastric cancer differs based on sex and anatomic location. *Gastroenterology* 2009; 137:824–833. 19445939 10.1053/j.gastro.2009.05.001 PMC3513767

[14] Li S Du H Wang Z Meta-analysis of the relationship between Epstein-Barr virus infection and clinicopathological features of patients with gastric carcinoma. *Sci China Life Sci* 2010; 53:524–530. 20596921 10.1007/s11427-010-0082-8

[15] Camargo MC Murphy G Koriyama C Determinants of Epstein-Barr virus-positive gastric cancer: an international pooled analysis. *Br J Cancer* 2011; 105:38–43. 21654677 10.1038/bjc.2011.215 PMC3137422

[16] Camargo MC Koriyama C Matsuo K Case-case comparison of smoking and alcohol risk associations with Epstein-Barr virus-positive gastric cancer. *Int J Cancer* 2014; 134:948–953. 23904115 10.1002/ijc.28402 PMC3961829

[17] Ryan JL Shen YJ Morgan DR Epstein-Barr virus infection is common in inflamed gastrointestinal mucosa. *Dig Dis Sci* 2012; 57:1887–1898. 22410851 10.1007/s10620-012-2116-5 PMC3535492

[18] Liberati A Altman DG Tetzlaff J The PRISMA statement for reporting systematic reviews and meta-analyses of studies that evaluate health care interventions: explanation and elaboration. *Ann Intern Med* 2009; 151:W65–W94. 19622512 10.7326/0003-4819-151-4-200908180-00136

[19] Tajima M Takeda F Takeshima T Human herpesviruses infections (I)-EBV infection and immunity in cancer patients. *Kansenshogaku Zasshi* 1991; 65:799–807. 1655918 10.11150/kansenshogakuzasshi1970.65.799

[20] Shibata D Weiss LM Epstein-Barr virus-associated gastric adenocarcinoma. *Am J Pathol* 1992; 140:769–774. 1314023 PMC1886378

[21] Shibata D Hawes D Stemmermann GN Epstein-Barr virus-associated gastric adenocarcinoma among Japanese Americans in Hawaii. *Cancer Epidemiol Biomarkers Prev* 1993; 2:213–217. 8391356

[22] Tokunaga M Land CE Uemura Y Epstein-Barr virus in gastric carcinoma. *Am J Pathol* 1993; 143:1250–1254. 8238241 PMC1887176

[23] Fukayama M Hayashi Y Iwasaki Y Epstein-Barr virus-associated gastric carcinoma and Epstein-Barr virus infection of the stomach. *Lab Invest* 1994; 71:73–81. 8041121

[24] Imai S Koizumi S Sugiura M Gastric carcinoma: monoclonal epithelial malignant cells expressing Epstein-Barr virus latent infection protein. *Proc Natl Acad Sci U S A* 1994; 91:9131–9135. 8090780 10.1073/pnas.91.19.9131 PMC44761

[25] Ott G Kirchner T Muller-Hermelink HK Monoclonal Epstein-Barr virus genomes but lack of EBV-related protein expression in different types of gastric carcinoma. *Histopathology* 1994; 25:323–329. 7835837 10.1111/j.1365-2559.1994.tb01350.x

[26] Shousha S Luqmani YA Epstein-Barr virus in gastric carcinoma and adjacent normal gastric and duodenal mucosa. *J Clin Pathol* 1994; 47:695–698. 7962618 10.1136/jcp.47.8.695 PMC502138

[27] Yuen ST Chung LP Leung SY In situ detection of Epstein-Barr virus in gastric and colorectal adenocarcinomas. *Am J Surg Pathol* 1994; 18:1158–1163. 7943537 10.1097/00000478-199411000-00010

[28] Anwar K Nakakuki K Imai H Infection of human papillomavirus (hpv) and epstein-barr-virus (ebv) and p53 overexpression in human gastric-carcinoma. *Int J Oncol* 1995; 7:391–397. 21552853 10.3892/ijo.7.2.391

[29] Harn HJ Chang JY Wang MW Epstein-Barr virus-associated gastric adenocarcinoma in Taiwan. *Hum Pathol* 1995; 26:267–271. 7890276 10.1016/0046-8177(95)90056-x

[30] Levine PH Stemmermann G Lennette ET Elevated antibody titers to Epstein-Barr virus prior to the diagnosis of Epstein-Barr-virus-associated gastric adenocarcinoma. *Int J Cancer* 1995; 60:642–644. 7860138 10.1002/ijc.2910600513

[31] Gulley ML Pulitzer DR Eagan PA Epstein-Barr virus infection is an early event in gastric carcinogenesis and is independent of bcl-2 expression and p53 accumulation. *Hum Pathol* 1996; 27:20–27. 8543306 10.1016/s0046-8177(96)90133-1

[32] Selves J Bibeau F Brousset P Epstein-Barr virus latent and replicative gene expression in gastric carcinoma. *Histopathology* 1996; 28:121–127. 8834519 10.1046/j.1365-2559.1996.287333.x

[33] Shin WS Kang MW Kang JH Epstein-Barr virus-associated gastric adenocarcinomas among Koreans. *Am J Clin Pathol* 1996; 105:174–181. 8607441 10.1093/ajcp/105.2.174

[34] Galetsky SA Tsvetnov VV Land CE Epstein-Barr-virus-associated gastric cancer in Russia. *Int J Cancer* 1997; 73:786–789. 9399652 10.1002/(sici)1097-0215(19971210)73:6<786::aid-ijc2>3.0.co;2-z

[35] Durmaz R Aydin A Koroglu M Investigation of the relationship between Epstein-Barr virus and ordinary gastric carcinoma using the nested polymerase chain reaction. *Acta Virol* 1998; 42:359–363. 10358740

[36] Hsieh LL Lin PJ Chen TC Frequency of Epstein-Barr virus-associated gastric adenocarcinoma in Taiwan. *Cancer Lett* 1998; 129:125–129. 9719452 10.1016/s0304-3835(98)00111-6

[37] Gurtsevich VE Galetskii SA Nered SN Detection and characterization of gastric carcinoma associated with epstein-barr herpes virus. *Vestn Ross Akad Med Nauk* 1999; 56–59. 10222834

[38] Kume T Oshima K Shinohara T Low rate of apoptosis and overexpression of bcl-2 in Epstein-Barr virus-associated gastric carcinoma. *Histopathology* 1999; 34:502–509. 10383694 10.1111/j.1365-2559.1999.00686.x

[39] Wan R Gao MQ Gao LY In situ detection of Epstein Barr virus in gastric carcinoma tissue in China high-risk area. *World J Gastroenterol* 1999; 5:531–532. 11819506 10.3748/wjg.v5.i6.531 PMC4688800

[40] Chapel F Fabiani B Davi F Epstein-Barr virus and gastric carcinoma in Western patients: comparison of pathological parameters and p53 expression in EBV-positive and negative tumours. *Histopathology* 2000; 36:252–261. 10692029 10.1046/j.1365-2559.2000.00843.x

[41] Corvalan A Koriyama C Akiba S Epstein-Barr virus in gastric carcinoma is associated with location in the cardia and with a diffuse histology: a study in one area of Chile. *Int J Cancer* 2001; 94:527–530. 11745439 10.1002/ijc.1510

[42] Lo YM Chan WY Ng EK Circulating Epstein-Barr virus DNA in the serum of patients with gastric carcinoma. *Clin Cancer Res* 2001; 7:1856–1859. 11448896

[43] Luqmani YA Linjawi SO Shousha S Detection of Epstein-Barr virus in gastrectomy specimens. *Oncol Rep* 2001; 8:995–999. 11496304 10.3892/or.8.5.995

[44] Kang GH Lee S Kim WH Epstein-barr virus-positive gastric carcinoma demonstrates frequent aberrant methylation of multiple genes and constitutes CpG island methylator phenotype-positive gastric carcinoma. *Am J Pathol* 2002; 160:787–794. 11891177 10.1016/S0002-9440(10)64901-2 PMC1867170

[45] Oda K Koda K Takiguchi N Detection of Epstein-Barr virus in gastric carcinoma cells and surrounding lymphocytes. *Gastric Cancer* 2003; 6:173–178. 14520531 10.1007/s10120-003-0247-2

[46] Ishii HH Gobe GC Yoneyama J Role of p53, apoptosis, and cell proliferation in early stage Epstein-Barr virus positive and negative gastric carcinomas. *J Clin Pathol* 2004; 57:1306–1311. 15563673 10.1136/jcp.2003.015081 PMC1770511

[47] Lopes LF Bacchi MM Elgui-de-Oliveira D Epstein-Barr virus infection and gastric carcinoma in Sao Paulo State, Brazil. *Braz J Med Biol Res* 2004; 37:1707–1712. 15517087 10.1590/s0100-879x2004001100016

[48] Lee MA Hong YS Kang JH Detection of Epstein-Barr virus by PCR and expression of LMP1, p53, CD44 in gastric cancer. *Korean J Intern Med* 2004; 19:43–47. 15053043 10.3904/kjim.2004.19.1.43 PMC4531546

[49] Wang Y Luo B Zhao P [Expression of Epstein-Barr virus genes in EBV-associated gastric carcinoma]. *Ai Zheng* 2004; 23:782–787. 15248912

[50] Alipov G Nakayama T Nakashima M Epstein-Barr virus-associated gastric carcinoma in Kazakhstan. *World J Gastroenterol* 2005; 11:27–30. 15609391 10.3748/wjg.v11.i1.27 PMC4205378

[51] Herrera-Goepfert R Akiba S Koriyama C Epstein-Barr virus-associated gastric carcinoma: Evidence of age-dependence among a Mexican population. *World J Gastroenterol* 2005; 11:6096–6103. 16273633 10.3748/wjg.v11.i39.6096 PMC4436624

[52] Luo B Wang Y Wang XF Expression of Epstein-Barr virus genes in EBV-associated gastric carcinomas. *World J Gastroenterol* 2005; 11:629–633. 15655811 10.3748/wjg.v11.i5.629 PMC4250728

[53] von Rahden BH Langner C Brucher BL No association of primary adenocarcinomas of the small bowel with Epstein-Barr virus infection. *Mol Carcinog* 2006; 45:349–352. 16493667 10.1002/mc.20163

[54] Koshiol J Qiao YL Mark SD Epstein-Barr virus serology and gastric cancer incidence and survival. *Br J Cancer* 2007; 97:1567–1569. 17987041 10.1038/sj.bjc.6604063 PMC2360259

[55] Saxena A Nath Prasad K Chand Ghoshal U Association of Helicobacter pylori and Epstein-Barr virus with gastric cancer and peptic ulcer disease. *Scand J Gastroenterol* 2008; 43:669–674. 18569983 10.1080/00365520801909660

[56] Kim Y Shin A Gwack J Epstein-Barr virus antibody level and gastric cancer risk in Korea: a nested case-control study. *Br J Cancer* 2009; 101:526–529. 19550421 10.1038/sj.bjc.6605146 PMC2720236

[57] Truong CD Feng W Li W Characteristics of Epstein-Barr virus-associated gastric cancer: a study of 235 cases at a comprehensive cancer center in U.S. *A J Exp Clin Cancer Res* 2009; 28:14. 19192297 10.1186/1756-9966-28-14 PMC2642773

[58] Chen JN Ding YG Feng ZY Association of distinctive Epstein-Barr virus variants with gastric carcinoma in Guangzhou, southern China. *J Med Virol* 2010; 82:658–667. 20166192 10.1002/jmv.21731

[59] Shukla SK Prasad KN Tripathi A Epstein-Barr virus DNA load and its association with Helicobacter pylori infection in gastroduodenal diseases. *Braz J Infect Dis* 2011; 15:583–590. 22218519

[60] de Aquino PF Carvalho PC Fischer JSD Epstein-Barr virus DNA associated with gastric adenocarcinoma and adjacent non-cancerous mucosa in patients from Manaus, Brazil. *Genet Mol Res* 2012; 11:4442–4446. 23096917 10.4238/2012.October.15.3

[61] Shukla SK Prasad KN Tripathi A Expression profile of latent and lytic transcripts of epstein-barr virus in patients with gastroduodenal diseases: a study from northern India. *J Med Virol* 2012; 84:1289–1297. 22711358 10.1002/jmv.23322

[62] Zhao J Jin H Cheung KF Zinc finger E-box binding factor 1 plays a central role in regulating Epstein-Barr virus (EBV) latent-lytic switch and acts as a therapeutic target in EBV-associated gastric cancer. *Cancer* 2012; 118:924–936. 21717425 10.1002/cncr.26184

[63] Yuan XY Wang MY Wang XY Non-detection of Epstein-Barr virus and Human Papillomavirus in a region of high gastric cancer risk indicates a lack of a role for these viruses in gastric carcinomas. *Genet Mol Biol* 2013; 36:183–184. 23885199 10.1590/S1415-47572013005000018 PMC3715283

[64] Martínez-López JL Torres J Camorlinga-Ponce M Evidence of Epstein-Barr virus association with gastric cancer and non-atrophic gastritis. *Viruses* 2014; 6:301–318. 24448220 10.3390/v6010301 PMC3917444

[65] Raab-Traub N Flynn K The structure of the termini of the Epstein-Barr virus as a marker of clonal cellular proliferation. *Cell* 1986; 47:883–889. 3022942 10.1016/0092-8674(86)90803-2

[66] Rowlands DC Ito M Mangham DC Epstein-Barr virus and carcinomas: rare association of the virus with gastric adenocarcinomas. *Br J Cancer* 1014-; 68:9. 10.1038/bjc.1993.472 PMC1968725 8217590

[67] Haas M Buttner M Rau TT Inflammation in gastric adenocarcinoma of the cardia: how do EBV infection, Her2 amplification and cancer progression influence tumor-infiltrating lymphocytes? *Virchows Arch* 2011; 458:403–411. 21359545 10.1007/s00428-011-1058-1

[68] Woo JR Liss MA Muldong MT Tumor infiltrating B-cells are increased in prostate cancer tissue. *J Transl Med* 2014; 12:30. 24475900 10.1186/1479-5876-12-30 PMC3914187

[69] Henle G Henle W Epstein-Barr virus-specific IgA serum antibodies as an outstanding feature of nasopharyngeal carcinoma. *Int J Cancer* 1976; 17:1–7. 175020 10.1002/ijc.2910170102

[70] de-Vathaire F Sancho-Garnier H de-The H Prognostic value of EBV markers in the clinical management of nasopharyngeal carcinoma (NPC): a multicenter follow-up study. *Int J Cancer* 1988; 42:176–181. 2841245 10.1002/ijc.2910420206

[71] Shinkura R Yamamoto N Koriyama C Epstein-Barr virus-specific antibodies in Epstein-Barr virus-positive and -negative gastric carcinoma cases in Japan. *J Med Virol* 2000; 60:411–416. 10686024 10.1002/(sici)1096-9071(200004)60:4<411::aid-jmv8>3.0.co;2-8

[72] Schetter AJ You WC Lennette ET Association of Epstein-Barr virus antibody levels with precancerous gastric lesions in a high-risk cohort. *Cancer Sci* 2008; 99:350–354. 18201267 10.1111/j.1349-7006.2007.00668.x PMC11159496

[73] Coghill AE Hildesheim A Epstein-Barr virus antibodies and the risk of associated malignancies: review of the literature. *Am J Epidemiol* 2014; 180:687–695. 25167864 10.1093/aje/kwu176 PMC4271109

